# Expression of Toll-Like Receptors 3, 7, and 9 in Peripheral Blood Mononuclear Cells from Patients with Systemic Lupus Erythematosus

**DOI:** 10.1155/2014/381418

**Published:** 2014-02-25

**Authors:** Agnieszka Klonowska-Szymczyk, Anna Wolska, Tadeusz Robak, Barbara Cebula-Obrzut, Piotr Smolewski, Ewa Robak

**Affiliations:** ^1^Department of Hematology, Medical University of Lodz, Ciolkowskiego 2, 93-510 Lodz, Poland; ^2^Copernicus Memorial Hospital, Pabianicka 62, 93-513 Lodz, Poland; ^3^Department of Experimental Hematology, Medical University of Lodz, Ciolkowskiego 2, 93-510 Lodz, Poland; ^4^Department of Dermatology and Venereology, Medical University of Lodz, ul. Plac J. Hallera 1, 90-647 Lodz, Poland

## Abstract

Systemic lupus erythematosus (SLE) is an autoimmune disease of unknown aetiology. The results of experimental studies point to the involvement of innate immunity receptors—toll-like receptors (TLR)—in the pathogenesis of the disease. The aim of the study was to assess the expression of TLR3, 7, and 9 in the population of peripheral blood mononuclear cells (PBMC) and in B lymphocytes (CD19^+^), T lymphocytes (CD4^+^ and CD8^+^) using flow cytometry. The study group included 35 patients with SLE and 15 healthy controls. The patient group presented a significantly higher percentage of TLR3- and TLR9-positive cells among all PBMCs and their subpopulations (CD3^+^, CD4^+^, CD8^+^, and CD19^+^ lymphocytes) as well as TLR7 in CD19^+^ B-lymphocytes, compared to the control group. There was no correlation between the expression of all studied TLRs and the disease activity according to the SLAM scale, and the degree of organ damage according to the SLICC/ACR Damage Index. However, a correlation was observed between the percentage of various TLR-positive cells and some clinical (joint lesions) and laboratory (lymphopenia, hypogammaglobulinemia, anaemia, and higher ESR) features and menopause in women. The results of the study suggest that TLR3, 7, and 9 play a role in the pathogenesis of SLE and have an impact on organ involvement in SLE.

## 1. Introduction

Systemic lupus erythematosus (SLE) is an autoimmune disease of connective tissue involving multiple organs. The pathogenesis of SLE is yet unknown. It is currently accepted that there are several genetic, environmental, and hormonal factors responsible for complex immunological disorders contributing to its development [1]. Recent studies have shown that abnormal stimulation of innate immunity may have a great influence on the immunopathogenesis of SLE. Hence, the receptors for Pathogen-Associated Molecular Patterns (PAMPs) have been the source of much recent attention.

One of the representatives of this group is toll-like receptors (TLRs). They are associated with innate immunity insofar as they are agents in the pathogenesis of SLE and lupus-like syndromes [2]. TLR expression has been revealed on various immune competent as well as nonimmune cells [3]. So far, 11 TLRs have been identified in humans. TLR3, TLR7, and TLR9 seem to be involved in the development of autoimmune diseases [4]. These receptors are located in the membrane of endosomes. Recognition of an appropriate PAMP takes place after its degradation in a lysosome. The ligation of a TLR activates a chain of proteins which transmit a signal to the nucleus, which in turn leads to increased production of proinflammatory cytokines, the expression of Major Histocompatibility Complex (MHC) class I and II antigens, and costimulatory molecules, which effectively activate antigen presentation and acquired immunity [5, 6]. Intracellular TLRs, apart from pathogen recognition and initiation of innate immunity, are capable of recognizing endogenous ligands [7]. In SLE patients, impaired apoptosis and invalid cell debris clearance lead to increased concentration of serum nucleic acids (ssRNA, dsRNA, and DNA), which are well-known ligands for TLR3, TLR7, and TLR9 [8].

Nucleic acid-dependent activation of endosomal TLR is mediated by BCR receptor on lymphocytes B and Fc*γ*, binding immunologic complexes and inducing their endocytosis [9].

The activation of these receptors by specific ligands is thought to initiate autoimmune processes [10], which has been confirmed by studies on animal SLE model. TLR stimulation leads to increased expression of proinflammatory cytokines (IL-6, IFN*α*, and TNF*α*), which may reflect the intensity of the disease. On the other hand, synthetic oligoDNA with TLR receptor inhibitory properties causes the opposite effect, leading to a clinical improvement being observed in animal SLE models [11].

The aim of our study was to assess the TLR3, TLR7, and TLR9 expression on peripheral blood mononuclear cells (PBMCs), including CD3^+^ T lymphocytes and their CD4^+^ and CD8^+^ subpopulations, and CD19^+^ B lymphocytes, in patients with SLE, compared to healthy controls. The original results of this study serve as the first presentation of a simultaneous analysis of the relationship between the expression of the studied TLRs and disease activity, the degree of organ damage, several clinical and laboratory parameters, and the influence of immunosuppressive treatment. Moreover, a correlation between the expression of TLRs and gender as well as pre- and postmenopausal period was evaluated.

## 2. Materials and Methods

### 2.1. Patients

Thirty-five SLE patients, diagnosed as having met at least 4 criteria according to the ACR, were included in the study [12]. All of the patients had been treated at the Department of Dermatology and Venereology, Medical University of Lodz and did not present symptoms of active infection or neoplastic disease at the time of the study. The study group comprised 30 women and 5 men aged from 25 to 65 years. The average duration of SLE was 7 years, ranging from 3 months to 21 years. Disease activity was assessed according to the SLAM (Systemic Lupus Activity Measure) scale [13]. The analysis included 24 clinical symptoms and 8 laboratory parameters. Patients who reached 10 and more points were diagnosed as having active SLE. During the study, 22 patients had active disease, and 13 were in remission.

Organ damage was then assessed with the SLICC/ACR (Systematic Lupus International Collaborating Clinics/American College of Rheumatology) Damage Index. Thirteen subjects received 0 points, which indicates no organ damage. However, 22 patients received ≥1 point and 8 of them ≥2 points, indicating severe organ damage. The average value according to the SLICC/ACR Damage Index was 1.09. The clinical and laboratory characteristics of our study group are depicted in Table 1. The control group included 15 healthy age- and gender-matched volunteers. All subjects included in the study gave their informed consent. The study was approved by the Bioethics Committee of the Medical University of Lodz.

### 2.2. Cell Isolation

Each sample contained twenty millilitres of the peripheral blood donated during routine laboratory examination. PBMCs were isolated by gradient centrifugation using Ficoll-Histopaque-1077 (PAA Laboratories, Pasching, Austria). Briefly, blood was precisely applied on the surface of the gradient and centrifuged at 1600 rpm for 20 min. The obtained buffy coat at the interphase was collected and dispersed in 5 mL of Hank's medium (Biomed, Lublin, Poland) and centrifuged at 1600 rpm for 10 min. The supernatant was collected and cells were washed twice with RPMI 1640 medium (PAA Laboratories, Pasching, Austria) at 1100 rpm for 5 min. each time. The PBMCs were then dispersed in phosphate buffered saline (PBS).

### 2.3. Assessment of TLR Expression in PBMCs

Isolated PBMCs were divided into 1 × 10^6^ cells per tube (each 100 *μ*L of PBS) and incubated with surface monoclonal antibodies against CD3, CD4, CD8, and CD19 conjugated with the fluorochromes allophycocyanin (APC), peridinin chlorophyll protein (Per-CP), and phycoerythrin-Cy7 (PE-Cy7) (all from BD Pharmingen, San Diego, CA, USA) at a concentration of 20 *μ*L/1 × 10^6^ cells, in darkness at room temperature for 30 min. The cells were then fixed and permeabilized using an intracellular TLR staining kit according to the producer's protocol (Imgenex, San Diego, CA, USA). The cells were then incubated with monoclonal antibodies against TLR3, TLR7, and TLR9 conjugated with fluorescein isothiocyanate (FITC) and phycoerythrin (PE) and their corresponding isotype controls (Invivogen, San Diego, CA, USA), at a concentration of 4 *μ*L/1 × 10^6^ cells, in darkness, at room temperature for 30 min. The cells were then washed in PBS and assessed using flow cytometry.

### 2.4. Flow Cytometry Analysis

**Figure 1:**
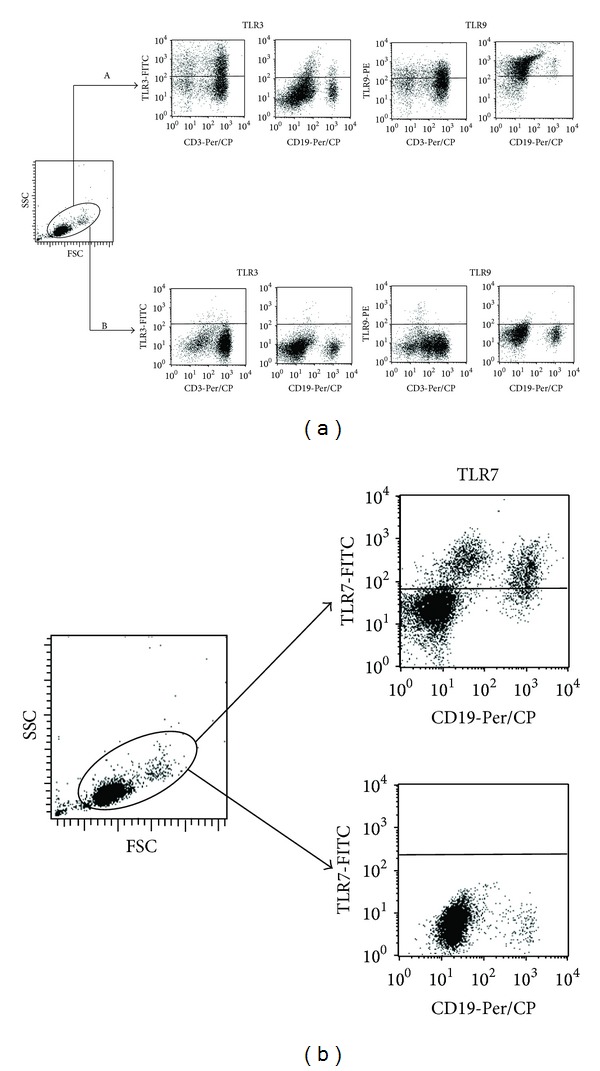
(a) Representative dot plots from flow cytometry measurements of TLR3 and TLR9 expression on T- and B-cells in patients (panel A) and healthy controls (panel B). (b) Representative dot plots from flow cytometry measurements of TLR7 expression on B-cells in patients (upper right dot plot) and healthy controls (lower right dot plot).

Six-color, two laser flow cytometry measurements were performed using the FACS Canto II cytometer, equipped with BD FACS Diva software (all Becton Dickinson, San Jose, CA, USA) as previously reported [14]. The cell fluorescence was estimated using standard fluorescence filters: FL1 (*λ* 313 nm ± 10), FL2 (*λ* 264 nm ± 10), FL3 (*λ* 374 nm ± 10) and FL4 (*λ* 467 nm ± 10), FL5 (*λ* 355 nm ± 10), and FL6 (*λ* 653 nm ± 10). For each sample, 10,000 events were analyzed. The lymphocyte population was discriminated from PBMCs by forward scatter (FSC) versus side scatter (SSC) distribution. Then, the percentages of CD3^+^, CD4^+^, CD8^+^, and CD19^+^ expressing TLR3, TLR7 or TLR9 were assessed. Finally, the ratios of TLR3, TLR7, and TLR9 in the whole population of PMBCs were calculated. Representative dot plots from flow cytometry measurements of TLR3 and TLR9 expression on T- and B-cells in patients and healthy controls (panel B) are presented in Figure 1(a). Representative dot plots from flow cytometry measurements of TLR7 expression on B-cells in patients and healthy controls are presented in Figure 1(b).

### 2.5. Statistical Analysis

For measurable characteristics, minimum and maximum values were shown; average values were calculated: the arithmetic mean, median, and mode were calculated as were the parameters describing the internal differentiation (standard deviation). The interquartile range was also calculated as the distance between the third and the first quartiles. For quality characteristics, the percentage of occurrence of the categories was determined.

To determine the pattern of distribution of the quantitative variables, the Shapiro-Wilk test was used. The Mann-Whitney test was used to assess the significance of any differences in average values between two groups, as the distribution pattern was not normal, and the ANOVA rank test and Kruskal-Wallis test, followed by a post hoc test of multiple comparisons of average ranks (Dunn test), were performed, to evaluate the differences in average values in several groups.

The assessment of the relationship between the measurable variables was based on the Spearman rank correlation coefficient. In all comparisons, the level of significance was *P* ≤ 0.05. Calculations were performed using STATISTICA v.9.1.

## 3. Results

### 3.1. Expression of TLR3, 7, and 9 in PBMCs, B and T Lymphocytes

**Figure 2:**
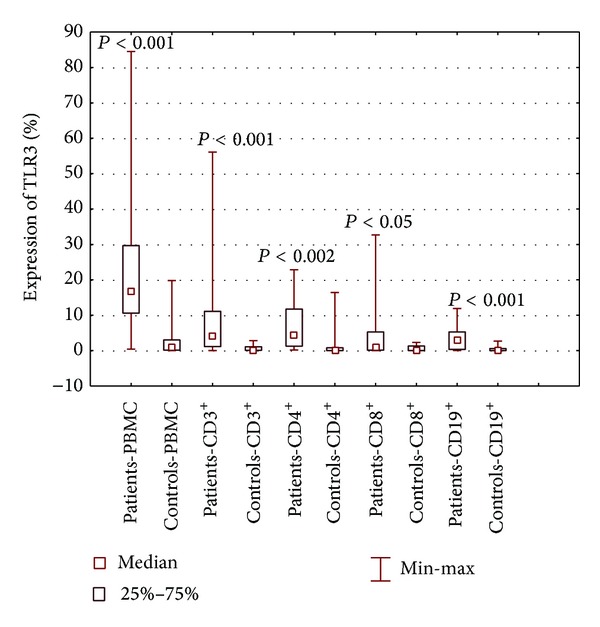
The percentage of PBMCs, lymphocytes B CD19^+^, and lymphocytes T CD3^+^, including CD4^+^ and CD8^+^, expressing TLR3 in patients with SLE and healthy controls.

**Figure 3:**
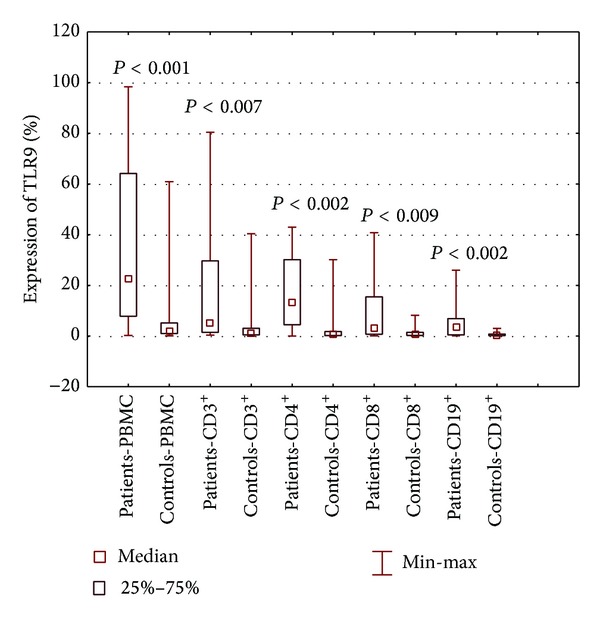
The percentage of PBMCs, lymphocytes B CD19^+^, and lymphocytes T CD3^+^, including CD4^+^ and CD8^+^, expressing TLR3 in patients with SLE and healthy controls.

**Figure 4:**
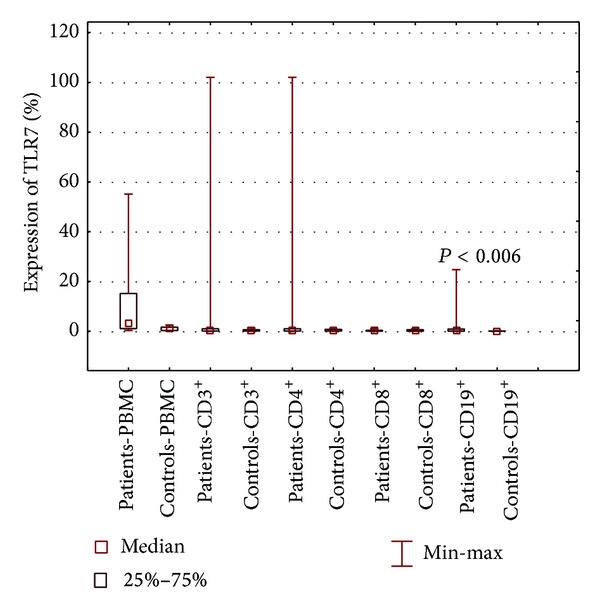
The percentage of PBMCs, lymphocytes B CD19^+^, and lymphocytes T CD3^+^, including CD4^+^ and CD8^+^, expressing TLR7 in patients with SLE and healthy controls.

Significantly higher percentages of TLR3- and TLR9-positive PBMCs and CD3^+^ T lymphocytes, including those positive for CD4 and CD8 antigens, as well as CD19^+^ B lymphocytes were observed among patients with SLE, compared to healthy controls (Figures 2 and 3). A higher percentage of CD19^+^ B lymphocytes expressing TLR7 was found in patients with SLE than in healthy subjects (*P* < 0.006) (Figure 4). With regard to PBMCs and both subpopulations of T lymphocytes, TLR7 expression did not differ between patients and healthy controls (Table 2).

### 3.2. TLR3, 7, and 9 Expression and SLAM Disease Activity

There were no significant correlations between the proportions of various cell subsets expressing the studied TLRs and disease activity (Table 2).

### 3.3. TLR3, 7, and 9 Expression and SLICC/ACR Damage Index

No statistically significant correlation was observed between the expression of any of the studied types of TLR among the given cell subpopulations and the degree of organ damage according to the SLICC/ARC Damage Index. However, subjects with severe organ dysfunction presented a higher percentage of TLR9-positive PBMCs, CD4^+^ and CD8^+^ T lymphocytes, and CD19^+^ B lymphocytes (Table 3).

### 3.4. Relationships between TLR Expression

A significant mutual correlation was seen to exist between the expression of TLR3 and TLR9 in PBMCs (*P* < 0.00001).

### 3.5. Correlation of TLR Expression with Gender

No statistically significant correlation was observed between the expression of studied TLRs and the patient's gender.

### 3.6. Correlation of TLR Expression with Pre- and Postmenopausal Period in Female Patients

A significantly higher percentage of CD19^+^ B lymphocytes expressing TLR7 was found in premenopausal women with SLE than in postmenopausal women (3.52% ± 6.46 versus 0.12% ± 0.17 resp., *P* < 0.03).

### 3.7. Correlation of TLR Expression with Clinical Findings and Laboratory Parameters

A significantly lower count of CD4^+^ cells with TLR9 was observed in patients with lymphopenia, compared with patients with a normal lymphocyte count (>1000/mm^3^) in the peripheral blood (4.59% ± 5.83 versus 6.86% ± 6.83 resp., *P* < 0.005).

In this subpopulation of cells, there was a significantly higher count of cells among patients with hypogammaglobulinemia, representing less than 12% of all proteins in the proteinogram analysis, compared to subjects with normal concentrations of gammaglobulins (32.12% ± 13.78 versus 11.46% ± 12.05, resp., *P* < 0.05).

Among patients with anaemia, there was a higher percentage of TLR7-positive CD3^+^  (4.19% ± 5.45), CD4^+^  (4.19% ± 5.45), and CD19^+^ cells (5.87% ± 8.71), compared to patients with haemoglobin concentration >12 g/dL (0.55% ± 1.08, *P* < 0.05; 0.55% ± 1.08, *P* < 0.03; 0.85% ± 2.24, *P* < 0.02, resp.).

Moreover, an erythrocyte sedimentation rate (ESR) of more than 25 was significantly more frequent in subjects with lower counts of TLR3-positive, CD19^+^ B lymphocytes compared to ESR ≤25 (2.55% ± 2.85 versus 5.10% ± 3.37, resp., *P* < 0.03).

A review of the clinical findings reveals that only patients with joint symptoms have lower TLR9-positive CD19^+^ B lymphocyte counts, compared to subjects with no joint symptoms. (4.59% ± 5.83 versus 6.86% ± 6.83, resp.; *P* < 0.005).

### 3.8. Correlation of TLR Expression with Immunosuppressive Treatments

No statistically significant correlation was observed between the expression of studied TLRs and immunosuppressive treatment.

## 4. Discussion

Despite intensive research in many centres, the pathogenesis of SLE remains poorly understood, and hence, the condition lacks targeted therapy. However, the discovery of TLRs in humans opened a new field in the studies of lupus [2], and our study of TLR3, TLR7, and TLR9 confirms their potential influence on the disease. TLR expression has been studied on the molecular level (mRNA), as well as the protein level, and involves many subsets of peripheral blood cells [15–20]. Higher expression of TLR9 has been shown in SLE patients, compared to healthy individuals, which is consistent with our findings. However, the results of studies concerning TLR3 and TLR7 expression are inconsistent. Most of them concentrate on TLR9 expression in B lymphocytes, probably due to the fact that these cells are the main source of pathological antibodies responsible for the propagation of the disease [16, 18, 20].

A higher count of CD19^+^ B and CD3^+^ T lymphocytes expressing TLR9 were seen in our study group, compared to healthy controls. This observation is similar to those obtained by Wu et al. [15], who assessed patients with newly diagnosed, untreated SLE. On the other hand, Papadimitraki et al. [16] observed a higher percentage of TLR9-positive CD19^+^ B lymphocytes in a group of patients with active disease, compared to those with inactive disease. What is more, they observed a decrease in TLR9 expression on B cells of as much as 50% when the patient entered remission. It is plausible that in remission, stimulation of B lymphocytes through TLR9 is less intense, and as a result of this phenomenon, the autoimmune inflammation subsides. A potential confirmation of this hypothesis is a study by Wong et al. [17], who demonstrated a positive correlation between the concentrations of proinflammatory cytokines and chemokines produced after TLR9 stimulation and disease activity. However, in our study, no difference was observed between active and inactive SLEs in terms of the percentage of TLR9-positive CD19^+^ B lymphocytes. This discrepancy between our and other centres may stem from the use of different criteria for patient selection. Patients with lupus nephritis dominated in the study by Papadimitraki et al. [16], constituting 36% of the whole study group, whereas they only constituted 3% of our group. Other research demonstrates greater TLR9 expression in the glomeruli of patients with lupus nephritis, and that the stimulation of glomeruli with endogenous TLR9 ligands augments inflammatory reactions in the kidneys [18].

Wong et al. [17] analysed TLR3, TLR7, and TLR9 expression in CD19^+^ B lymphocytes and CD4^+^ and CD8^+^ T lymphocytes among 16 Chinese women. This is the only available publication which addressed the same markers as the present study. The results regarding TLR3 and TLR9 expression in CD19^+^ B lymphocytes and CD4^+^ and CD8^+^ T lymphocytes obtained in by both the present study and that of Wong et al. [17] are similar. However, these results need to be confirmed by RT PCR on T cells as was done on B cells by Nakano et al. [20]. Differences between those results concerned only TLR7 population. While our report shows a markedly higher count of TLR7-positive lymphocytes B CD19^+^ in SLE patients than in healthy subjects, Wong et al. [17] did not find any difference in TLR7 expression for any cell subset between patients and healthy controls. However, after TLR7 stimulation, they observed an increase in the production of the chemokines CXCL10 and CCL5 by PBMCs from patients with SLE. The observed inconsistence of the results may be due to heterogeneous nature of the study groups used by the two studies or their different genetic background. Similar to our results, although obtained via molecular techniques, are the findings by Komatsuda et al. [19], who report that the concentrations of mRNA for TLR7 and TLR9 in PBMCs are significantly higher among patients than in healthy controls.

There are several publications regarding correlations between the expression of TLRs with SLE activity, but the conclusions are contradictory. Wong et al. [17] do not report any such correlation in terms of TLR3, TLR7, or TLR9 expression. The lack of any relationship was probably due to the relative predominance of subjects with an inactive disease, according to SLEDAI scale (SLE Disease Activity Index).

In addition, no such significant relationship was demonstrated, although the majority of patients (63%) presented with active SLE. Nakano et al. [20] studied 19 subjects in the active SLE phase and identified a positive relationship between TLR9 MFI (Mean Fluorescence Intensity) in B lymphocytes and SLEDAI score. Wu et al. [15] analysed a group of 35 newly diagnosed patients and found a negative correlation between the percentage of TLR9-positive B cells and SLE activity. They pointed to a possible protective role of TLR9 in the development and propagation of SLE. The discrepancy of published data may be explained by heterogeneous study groups in terms of clinical and therapeutic parameters.

The presence of anti-dsDNA antibodies and TLR expression was also noted in the present study. This type of antibody is a pathognomonic marker of SLE, specific for renal involvement. Available publications concerning the relationship between the expression of TLR and the presence of anti-dsDNA antibodies are inconsistent. There was a positive correlation between the concentration of anti-dsDNA antibodies with the percentage of TLR9-positive CD19^+^ B lymphocytes from patients with active SLE [16]. However, other studies, as well as our own results, do not reveal any significant relationships between these parameters [17, 21]. On the contrary, Komatsuda et al. [19] observed a negative correlation between anti-dsDNA autoantibodies and the TLR9 mRNA content in cells. This may be explained by the heterogeneity of studied populations in terms of clinical presentation, accompanying diseases, treatment modalities, and occult infections, in particular. Komatsuda et al. [19], unlike other researchers, evaluated an entire PBMC population, including B and T lymphocytes and monocytes, and subjects included in the study were untreated. Moreover, while the authors assessed the concentration of anti-dsDNA antibodies, the others only noted their presence.

A significant part of our study was the assessment of TLR expression with characteristic clinical and laboratory parameters. In the present study, a lower percentage of CD4^+^ cells expressing TLR9 was seen in patients with lymphopenia, compared to those with lymphocyte counts above 1000/*μ*L. To our knowledge, there has been only one publication evaluating the relationship between lymphocyte count and TLR expression so far. Komatsuda et al. [19] did not find any relationship between the amounts of TLR2-5, TLR7, and TLR9 mRNA in PBMCs and leukocyte, lymphocyte, neutrophil, and platelet counts, although 18 of 21 subjects presented with hematological abnormalities.

It may be plausible that the decreased lymphocyte count of CD4^+^ T lymphocytes coexpressing TLR9 may be related to immunosuppressive treatment in this group of patients. This hypothesis is confirmed in a study by Lu et al. [22], who report that methylprednisolone inhibits the survival of activated CD4^+^ lymphocytes activated by specific TLR3 and TLR9 ligands in vitro but has no effect on their expression. What is more, immunosuppressive therapy leads to hypogammaglobulinemia and secondarily induces immune deficiency [23]. There were 8 subjects (23%) with hypogammaglobulinemia in our group and half of them were receiving immunosuppressants. All our patients were in the active stage of SLE. They presented with a significantly higher percentage of CD4^+^ TLR9-positive cells, compared to individuals with gammaglobulin levels above 12%. It may be that the treatment with glucocorticosteroids and/or cytostatic agents led to a decrease of gammaglobulins but did not diminish the number of CD4^+^ T lymphocytes expressing TLR9. This may be due to a low number of subjects with hypogammaglobulinemia with and without immunosuppressive treatment. However, it cannot be excluded that the differences in TLR9 expression between these two subgroups (lymphopenia and hypogammaglobulinemia) may be caused by the different numbers of patients receiving immunosuppressive drugs, the number being lower in the case of hypogammaglobulinemia.

Our findings warrant further studies on TLR expression in T lymphocytes from patients with SLE, as they may lead to a better understanding of the complex interactions between innate and acquired immunity in the pathogenesis of SLE. One profitable course of action would be to inquire into the molecular level of the cell cycle using RT-PCR. The results of the present study note a lower count of CD19^+^ B lymphocytes with TLR3 in patients with ESR >25. Higher ESR reflects the presence of inflammatory process in the body.

Glucocorticosteroids have a strong anti-inflammatory potential, caused by the inhibition of cytokine biosynthesis at the genome level. They also interfere with the intracellular signaling pathways of cytokines [24]. Treatment with this group of drugs may have led to a decrease in cytokines in the sera of patients with high ESR, resulting in a lower percentage of B cells expressing TLR3. Despite this, the treatment did not quench the inflammatory process and, therefore, did not lower the increased ESR.

There are few publications concerning the expression of TLR3 and ESR. The only available article by Nakano et al. [20], where the authors evaluated the correlation between TLR9s in lymphocytes B and T with increased ESR, does not confirm any significant relationship.

A higher count of CD3^+^, CD4^+^, and CD19^+^ cells coexpressing TLR7 was found in patients with anemia compared to subjects with hemoglobin above 12 g/dL. In SLE, anemia may stem from autoimmune hemolysis or chronic inflammatory process (Anemia of Chronic Diseases, ACD). In our group, ACD was seen in 25% of patients. This type of anaemia develops due to a chronic inflammatory reaction, characterized by increased concentrations of TNF-*α*, IL-1, or IFN-gamma, which inhibit the secretion of erythropoietin and availability of iron, essential for efficient erythropoiesis [25].

As a result of TLR activation, numerous proinflammatory cytokines are expressed, including these responsible for SLE development. It was indicated that proinflammatory cytokines may regulate TLR expression [26]. Moreover, the proinflammatory cytokine-dependent expression of TLR, adaptor proteins, and kinases participating in signal transduction towards the cell interior has been proved [27]. An increased concentration of IFN-*α* in the serum of SLE patients, combined with raised IFN-type I dependent gene expression in the mononuclear cells of peripheral blood cells, has been characterized as interferon signature [28]. The continuous, TLR-mediated biosynthesis of IFN-*α* by nucleic acids containing immunologic complexes may be responsible for the interferon signature phenomenon. Moreover, it has been revealed that the level of IFN alpha-dependent gene expression is correlated with SLE activity and more detrimental clinical disease forms, associated with damage to the kidneys, bone marrow, or cells of the central nervous system [29, 30].

Increased INF-alpha concentration is regarded as the response to the continuous activation of TLR pathways [31]. However, Komatsuda et al. [19] did not confirm any correlation between TLR and IFN-alpha induced *LY6E* (lymphocyte antigen 6 complex, locus E) gene expression.

The observed higher percentage of CD3^+^, CD4^+^, and CD19^+^ cells with TLR7 among subjects with anemia may reflect the presence of chronic inflammation and increased proinflammatory cytokines. In our study group, a higher count of TLR7-positive B and T cells was seen although 78% of patients received immunosuppressive drugs. This may be due to the majority of patients experiencing active SLE (89%).

Hormonal factors play an important role in the development of SLE. Exacerbation of SLE may be induced by the usage of oestrogen-based anticontraceptive pills, that may also elevate the risk of a more severe disease course [32]. However, during menopause SLE tends to become milder, which is probably due to a decrease in oestrogen levels in peripheral blood [33]. In our study, we demonstrated a statistically significant higher percentage of B lymphocytes CD19^+^ expressing TLR7 in premenopausal women, compared to females after menopause. These observations suggest the influence of female sex hormones on TLR7 expression on lymphocytes B. This effect has been confirmed in other studies. Young et al. (2011) indicated the increased in vitro expression of endosomal TLRs, including TLR7, on PBMC cells from normal women ader estradiol stimulation, with no effect after treatment with testosteron [34]. In another study, 17*β*-estradiol treatment of normal postmenopausal women enhanced TLR7/9 pDC production of IFN*α* [35]. However, secretion of IFN*α* by plasmacytoid dendritic cells after TLR7 activation was lower in postmenopausal than in premenopausal females [36]. Furthermore, stimulation of TLR7 with a synthetic agonist in lupus-prone mice lacking the alpha oestrogen receptor led to a lower IL-6 synthesis by lymphocytes B than in wild type animals [37].

When the clinical symptoms were analyzed, a significantly lower count of lymphocytes B CD19^+^ with TLR9 was found in patients with joint symptoms (75% of subjects) than in patients with no joint symptoms (25%). Some publications describe the expression of TLRs in rheumatoid arthritis [38, 39] and note that patients demonstrate higher expression of TLR2, 3, and 4 on fibroblasts from the synovial tissue. The synovial fluid contains various TLR ligands such as peptidoglycan, dsRNA released from necrotic cells, lipopolysaccharides, and CpG-rich nucleic acid. Their presence stimulates the synthesis of many proinflammatory cytokines and chemokines, which sustain inflammation in joints [38, 39]. The lower percentage of CD19^+^ B lymphocytes expressing TLR9 in patients with joint symptoms in our study group may be related to the presence of immunosuppressive treatment. However, immunosuppressive therapy was found to have no influence on TLR expression in our study, which is consistent with the results of other researchers [16, 17]. Only one study contradicts this: Nakano et al. [20] reports a significant decrease of MFI for TLR9 in CD20^+^ B lymphocytes in 8 out of 11 patients with SLE.

No correlation was observed between TLR expression and the degree of organ damage, according to SLICC/ACR. The lack of any relationship may be explained by the fact that the organ damage reflects the final outcome of the inflammatory process. Therefore, it is no longer active and the cellular interactions are less pronounced. So far, there has been no research concerning these findings. A significant positive correlation was recorded between TLR3 and TLR9 expression in PBMCs (*P* < 0.00001). Unfortunately, no publication regarding mutual TLR relations can be found. However, it is probable that the significant correlation of TLR3 and TLR9, but not TLR7, stems from the higher lability of ssRNA (the ligand for TLR7), which undergoes rapid degradation by ribonucleases and is quickly removed from circulation.

TLRs are able to recognise endogenous antigens which are released upon cell damage or stress and have been shown to play a key role in numerous autoimmune diseases [40, 41]. These TLR ligands bind TLRs, possibly initiate intracellular signaling pathways, and may initiate autoimmunity processes. TLRs act on the monocyte-macrophage system and activate dendritic cells, which then engage self-antigens, as the first step for the induction of autoimmunity [41]. TLR2 has been shown to induce the development of Th17 cells in vivo [42]. In addition, IL-17 and IFN-*γ* appear to act synergistically in causing autoimmune processes [43]. TLR9 activation induces the expression of membrane-bound B-cell activating factor (BAFF) on human B cells and leads to increased proliferation in response to both soluble and membrane-bound BAFF [44]. A sizable body of evidence suggests that the endolysosome-restricted nucleic acid sensing subset of TLRs (NA-TLRs) plays an important role in the production of antinuclear autoantibodies [45]. Recently, Koh et al. [46] documented that NA-TLRs promote the induction of antinuclear Abs in SLE. Their data indicates that the presence of NA-TLRs in B cells is necessary to drive the initial autoimmune response and to promote the activation and escape of tolerance of self-reactive B cells. In addition, overexpression of TLR7 within the B cell compartment was found to enhance B cell TLR7 expression, permit the specific development of anti-RNA autoantibody production, and exacerbate SLE disease in an animal model [47]. Moreover, the inhibition of both TLR7 and TLR9 reduces autoimmune pathology in experimental SLE [48, 49]. This observation suggests that the aberrant activation of a number of TLR pathways may lead to the initiation and/or perpetuation of SLE and may indicate the direction for more specific therapy of this disease.

In conclusion, our results suggest that TLRs exert an influence on SLE development and describe the potential roles played by TLRs in the involvement of specific organs in this disease. Even so, more targeted studies concerning the biology and function of TLRs are warranted and may lead to the development of a new class of drugs.

## Figures and Tables

**Table 1:** Clinical and laboratory characteristics of the study group.

Features	Number of patients (%) or mean (range)	%
Nunber of patients	35 (100%)	100%
Age (years)	43.6 (25–65)	
Disease duration (years)	7.16 (0.25–21)	
Gender (male/female)	5 (14.3)/30 (85.7)	14.3/85.7
Active/nonactive SLE (SLAM)	22 (62.8)/13 (37.2)	62.8/37.2
SLICC/ACR-0	13 (37.2)	37.2
SLICC/ACR-1	14 (40)	40
SLICC/ACR-2	5 (14.3)	14.3
SLICC/ACR-3	1 (2.8)	2.8
SLICC/ACR-4	1 (2.8)	2.8
SLICC/ACR-5	1 (2.8)	2.8
Immunosuppressive therapy Y/N	19 (54.3)/16 (45.7)	54.3/45.7
Joint symptoms	25 (71.4)	71.4
Skin lesions	34 (97.1)	97.1
Reticuloendothelial system involvement	10 (28.6)	28.6
Cardiovascular symptoms	22 (62.8)	62.8
Neurological symptoms	29 (82.8)	82.8
Renal symptoms (creatinine > 1.3 mg/dL)	1 (2.8)	2.8
Anaemia (Hb < 12 g/dL)	9 (25.7)	25.7
Leucopenia (WBC < 3.5 G/L)	9 (25.7)	25.7
Lymphopenia (lymphocyte count < 1 G/L)	15 (42.8)	42.8
Low platelet count (PLT < 150 G/L)	13 (37.1)	37.1
Erythrocyte sedimentation rate (ESR > 25 mm/h)	22 (62.8)	62.8
Gammaglobulins < 12% all proteins	8 (22.8)	22.8
Antinuclear antibodies (ANA > 1/160)	34 (97.1)	97.1
The presence of anti-dsDNA antibodies	9 (25.7)	25.7
Complement C3 < 0.9 G/L	18 (51.4)	51.4
Complement C4 < 0.1 G/L	7 (20)	20.0

SLICC/ACR: Systematic Lupus International Collaborating Clinics/American College of Rheumatology; SLAM: Systemic Lupus Activity Measure.

**Table 2:** The percentage of TLR3-, TLR7-, and TLR9-positive PBMCs and lymphocytes B CD19^+^ and lymphocytes T CD3^+^, including CD4^+^ and CD8^+^, in patients with SLE (active and nonactive), compared to healthy controls.

Cell subpopulation (% positive cells)	Patients inactive *n* = 14 (a)	Patients active *n* = 21 (b)	Healthy *n* = 15 (c)	Statistical significance
TLR3 in PBMC				
$\overset{-}{x}\pm \text{SD}$	24.01 ± 25.06	23.35 ± 17.18	2.58 ± 4.97	(a)–(c) *P* < 0.002
Range	(0.70–84.50)	(0.43–61.83)	(0.00–19.80)	(b)-(c) *P* < 0.001
TLR9 in PBMC				
$\overset{-}{x}\pm \text{SD}$	33.72 ± 31.45	36.36 ± 32.67	1.09 ± 0.75	(a)–(c) *P* < 0.001
Range	(0.98–97.5)	(0.3–98.37)	(0.05–2.50)	(b)-(c) *P* < 0.001
TLR3 in CD3				
$\overset{-}{x}\pm \text{SD}$	12.34 ± 18.72	8.69 ± 11.13	0.69 ± 0.94	(a)–(c) *P* < 0.002
Range	(0.13–56.09)	(0.0–39.22)	(0.00–2.79)	(b)-(c) *P* < 0.004
TLR3 in CD4				
$\overset{-}{x}\pm \text{SD}$	8.27 ± 9.59	6.51 ± 7.05	1.56 ± 4.32	(a)–(c) *P* < 0.002
Range	(0.23–22.86)	(0.18–22.60)	(0.00–16.43)	(b)-(c) *P* < 0.02
TLR3 in CD8				
$\overset{-}{x}\pm \text{SD}$	7.46 ± 14.28	3.32 ± 4.15	0.58 ± 0.78	(a)–(c) *P* > 0.05
Range	(0.1–32.71)	(0.01–12.3)	(0.00–2.30)	(b)-(c) *P* > 0.05
TLR3 in CD19				
$\overset{-}{x}\pm \text{SD}$	3.46 ± 3.93	3.54 ± 2.81	0.41 ± 0.72	(a)–(c) *P* < 0.02
Range	(0.04–11.90)	(0.03–9.19)	(0.00–2.70)	(b)-(c) *P* < 0.001
TLR7 in CD19				
$\overset{-}{x}\pm \text{SD}$	1.41 ± 2.89	2.85 ± 6.52	0.05 ± 0.06	(a)–(c) *P* > 0.05
Range	(0.00–9.80)	(0.00–24.73)	(0.00–0.20)	(b)-(c) *P* < 0.05
TLR9 in CD3				
$\overset{-}{x}\pm \text{SD}$	17.78 ± 25.68	18.95 ± 26.46	4.39 ± 10.15	(a)–(c) *P* > 0.05
Range	(0.36–66.90)	(0.37–80.44)	(0.00–40.34)	(b)-(c) *P* < 0.03
TLR9 in CD4				
$\overset{-}{x}\pm \text{SD}$	15.43 ± 15.87	18.44 ± 16.04	3.14 ± 7.85	(a)–(c) *P* > 0.05
Range	(1.27–34.65)	(0.04–42.96)	(0.00–30.18)	(b)-(c) *P* < 0.02
TLR9 in CD8				
$\overset{-}{x}\pm \text{SD}$	12.06 ± 17.18	7.04 ± 7.88	1.20 ± 2.04	(a)–(c) *P* > 0.05
Range	(0.74–40.77)	(0.08–21.94)	(0.00–8.17)	(b)-(c) *P* > 0.05
TLR9 in CD19				
$\overset{-}{x}\pm \text{SD}$	4.33 ± 5.86	5.84 ± 6.35	0.78 ± 0.95	(a)–(c) *P* > 0.05
Range	(0.10–22.00)	(0.10–26.00)	(0.00–3.00)	(b)-(c) *P* < 0.005

**Table 3:** The percentage of TLR9-positive PBMCs, lymphocytes B CD19^+^, and lymphocytes T CD3^+^ in relation to SLICC/ACR organ damage.

Cells subpopulations (% positive cells)	SLICC = 0 *n* = 13 (a)	SLICC = 1 *n* = 14 (b)	SLICC = 2 *n* = 5 (c)	Healthy *n* = 15 (d)	Statistical significance
TLR9 in PBMC					
$\overset{-}{x}\pm \text{SD}$	31.04 ± 31.93	35.02 ± 33.89	58.79 ± 27.01	1.09 ± 0.75	(a)–(d) *P* < 0.003
Median	13.92	27.30	64.40	1.10	(b)–(d) *P* < 0.001
Range	(0.72–97.5)	(0.3–98.37)	(27.75–93.53)	(0.05–2.50)	(c)-(d) *P* < 0.001
TLR9 in CD3					
$\overset{-}{x}\pm \text{SD}$	14.47 ± 21.71	22.88 ± 32.12	28.81 ± 26.21	3.14 ± 7.85	
Median	4.26	1.98	23.58	0.75	
Range	(0.36–66.9)	(0.4–80.44)	(5.37–62.73)	(0.00–30.18)	
TLR9 in CD19					
$\overset{-}{x}\pm \text{SD}$	2.69 ± 2.19	6.04 ± 7.59	9.95 ± 7.62	0.78 ± 0.95	(c)-(d) *P* < 0.006
Median	3.25	3.02	10.29	0.45
Range	(0.1–6.52)	(0.10–26.00)	(3.32–22.0)	(0.00–3.00)

$\overset{-}{x}$: mean; SD: standard deviation.

The bold font emphasises that the higher SLICC value is parallelled with the higher percentage of PBMCs, lymphocytes CD3+ and CD19+ with TLR9.
